# Measuring site fidelity and spatial segregation within animal societies

**DOI:** 10.1111/2041-210X.12751

**Published:** 2017-03-20

**Authors:** Thomas O. Richardson, Luca Giuggioli, Nigel R. Franks, Ana B. Sendova‐Franks

**Affiliations:** ^1^ Department of Ecology and Evolution University of Lausanne Lausanne Switzerland; ^2^ Department of Engineering Design and Mathematics University of the West of England Bristol UK; ^3^ Bristol Centre for Complexity Sciences University of Bristol Bristol UK; ^4^ Department of Engineering Mathematics University of Bristol Bristol UK; ^5^ School of Biological Sciences University of Bristol Bristol UK

**Keywords:** animal movement, ant, non‐Markov, random walk, social network, social insect, *Temnothorax albipennis*

## Abstract

1. Animals often display a marked tendency to return to previously visited locations that contain important resources, such as water, food, or developing brood that must be provisioned. A considerable body of work has demonstrated that this tendency is strongly expressed in ants, which exhibit fidelity to particular sites both inside and outside the nest. However, thus far many studies of this phenomena have taken the approach of reducing an animal's trajectory to a summary statistic, such as the area it covers.
2. Using both simulations of biased random walks, and empirical trajectories from individual rock ants, *Temnothorax albipennis*, we demonstrate that this reductive approach suffers from an unacceptably high rate of false negatives.
3. To overcome this, we describe a site‐centric approach which, in combination with a spatially‐explicit null model, allows the identification of the important sites towards which individuals exhibit statistically significant biases.
4. Using the ant trajectories, we illustrate how the site‐centric approach can be combined with social network analysis tools to detect groups of individuals whose members display similar space‐use patterns.
5. We also address the mechanistic origin of individual site fidelity; by examining the sequence of visits to each site, we detect a statistical signature associated with a self‐attracting walk – a non‐Markovian movement model that has been suggested as a possible mechanism for generating individual site fidelity.

## Introduction

The phenomenon of ‘recurrence’, in which the movement of an individual is biased towards a set of previously visited locations, is widespread in the animal kingdom (Gonzalez, Hidalgo & Barabasi 2008; Boyer, Crofoot & Walsh 2011; Schreier & Grove 2014). Recent work comparing the mobility patterns of humans and vervet monkeys has shown that recurrence is a fundamental statistical property common to both (Boyer, Crofoot & Walsh 2011). In humans, examples of important locations at which recurrence is most strongly expressed include homes, workplaces, restaurants and the transit routes that connect them (Sun *et al*. 2013). In other non‐human animals these locations might take the form of watering holes, foraging patches, leks, or nesting areas where there are brood that must be provisioned regularly.

Depending upon the study system and the context, preferential bias towards previously visited locations has been variously labelled recurrence (Gonzalez, Hidalgo & Barabasi 2008; Song *et al*. 2010; Boyer, Crofoot & Walsh 2011), recursion (Bar‐David *et al*. 2009; Benhamou & Riotte‐Lambert 2012; Fagan *et al*. 2013; Berger‐Tal & Bar‐David 2015), site tenacity (Hahn & Maschwitz 1985), site allegiance (Dejean & Turillazzi 1992), site recognition (Salo & Rosengren 2001), site fidelity (Lamb & Ollason 1994; Schwarzkopf & Alford 2002; Giuggioli & Bartumeus 2012), spatial fidelity (Sendova‐Franks & Franks 1995), ‘ortstreue’ (Rosengren & Fortelius 1986) and route fidelity (Rosengren 1971). Recursive movement has been particularly well documented in the social insects – ants, bees, wasps and termites – where the phenomenon is most often referred to as site fidelity. Social insects show strong site fidelity both outside the nest (Traniello, Fourcassié & Graham 1991; Fourcassié & Traniello 1994; Lamb & Ollason 1994; Schatz, Lachaud & Beugnon 1995; Beverly *et al*. 2009; Salo & Rosengren 2001), and within it (Seeley 1982; Sendova‐Franks & Franks 1995; Jandt & Dornhaus 2009; Frohschammer & Heinze 2009; Baracchi *et al*. 2010; Jeanson 2012). For example, wood ant workers show a strong tendency to re‐use one of the multiple foraging trunk‐trails emanating from the nest mound (Rosengren , 1971, 1977), a preference that can persist over several seasons (Rosengren 1971). Inside the nests of ants and bees, there is a strong division of labour, in which work is divided into discrete tasks that are spatially segregated into different zones, with each zone being populated by a particular set of worker task specialists (Seeley 1982; Mersch, Crespi & Keller 2013; Baracchi & Cini 2014). This division of labour is thought to increase colony productivity, and has led to social insects being ecologically dominant in many ecosystems (Oster & Wilson 1978). Hence the origin and quantification of individual spatial fidelity have been and continue to be, of considerable interest to scientists interested in the organization of animal societies. Here we study site fidelity in colonies of the rock ant, *Temnothorax albipennis*. We chose this species because the workers exhibit site fidelity within the nest (Sendova‐Franks & Franks 1993), and because the nearly two‐dimensional geometry of natural rock ant nests – flat cavities between rock layers – makes them ideal for studies of spatial movement.

A variety of methods is now available for identifying different spatio‐temporal components of site fidelity. For example, there has been a recent growth in methods for identifying routine movement patterns, such as periodic returns to previously‐visited locations (Bar‐David *et al*. 2009; Riotte‐Lambert, Benhamou & Chamaillé‐Jammes 2013; Péron *et al*. 2016), or repetitive sequences of visits to particular locations (Riotte‐Lambert, Benhamou & Chamaillé‐Jammes 2017). Similarly, there are several tools to evaluate whether there is a stable home range over which the animal typically roams, or a core area to which it frequently returns, such as comparing the degree of spatial overlap between consecutive time periods (Cooper 1978; Van Beest *et al*. 2013), or checking whether the time‐series of the total area that the animal covers (Van Moorter *et al*. 2009), or its net displacement (Börger, Dalziel & Fryxell 2008), saturate over time. Despite this plethora of techniques, many studies of within‐nest site fidelity in social insects still adopt a ‘reductive’ approach in which a complex spatio‐temporal object – an animal trajectory – is aggregated over time and space into a single summary statistic such as the area the trajectory covers (Jandt & Dornhaus 2009; Baracchi *et al*. 2010; Baracchi & Cini 2014). This preference may be derived from the nest wall severely circumscribing individual movement; as the total area covered and the net displacement of a physically bounded random walk both saturate over time, it is difficult for the above methods to distinguish between an agent that moves randomly within the nest, and one that has a preference for one (or several) parts of the nest. Hence, the primary motivation for the current study is to provide an analytical framework that can identify those individuals that exhibit site fidelity that can pinpoint the sites to which they are loyal, and that is robust to the presence of physical boundaries. The second motivation stems from the fact that existing measures of spatial fidelity are often based upon descriptions of the space use patterns of individuals (Sendova‐Franks & Franks 1993; Frohschammer & Heinze 2009; Baracchi *et al*. 2010; Benhamou & Riotte‐Lambert 2012; Mersch, Crespi & Keller 2013) or groups Baracchi & Cini (2014), rather than upon quantitative comparisons between the observed pattern and an absolute standard (but see Sendova‐Franks & Franks 1995; Jandt & Dornhaus 2009). In other words, rigorous hypothesis‐testing, involving comparisons between the observation and the expectation under the assumption of random movement, as predicted by a mathematical or statistical null model, has sometimes been lacking.

In the first part of the paper, we present an extension of a recent site‐centric framework which has been developed for the analysis of human digital mobility traces (Crandall *et al*. 2010; Sun *et al*. 2013) and animal movement ecology (Boyer, Crofoot & Walsh 2011; Benhamou & Riotte‐Lambert 2012; Lyons, Turner & Getz 2013; Fagan *et al*. 2013; Berger‐Tal & Bar‐David 2015). Contrary to the traditional reductive approach in which the trajectory is reduced to a single summary statistic, in the site‐centric framework space is discretized into a regular grid, and the visitation statistics of a given individual for each site are analysed independently. In our extension, we demonstrate that sites to which individuals exhibit positive or negative biases can be identified by comparing these site‐visitation statistics with an absolute standard, provided by null model synthetic trajectories that exhibit no spatial biases. Further, using both simulations of biased random walks, and empirical analysis of ant trajectories, we show that this combined framework is more sensitive at identifying individuals that exhibit site fidelity than the traditional reductive approach.

Whilst our understanding of the social organization of colonies of ants (Sendova‐Franks *et al*. 2010; Blonder & Dornhaus 2011; Jeanson 2012), bees (Naug & Smith 2007; Otterstatter & Thomson 2007), and other highly social species (Williams & Lusseau 2006; Drewe 2010), has been greatly advanced by the application of tools from network science, these tools are only just beginning to be applied to the spatial organization of these societies (see e.g. Mersch, Crespi & Keller 2013; Baracchi & Cini 2014; Richardson & Gorochowski 2015). Therefore, in the second part of the paper, we use the results of the site‐centric analysis of the ant trajectories to construct spatial networks in which each edge represents the spatial overlap in the site‐visitation patterns of two ants. We then show how modern network partitioning methods can be used to identify groups of ants with distinctive space use patterns.

Recent theoretical modelling has shown that biologically interesting behaviours, such as the establishment of a territory, core area, or home range, can emerge when an individual's movement decisions are influenced by its historical movement patterns (Van Moorter *et al*. 2009; Foster, Grassberger & Paczuski 2009; Spencer 2012; Fagan *et al*. 2013; Boyer & Solis‐Salas 2014; Berger‐Tal & Bar‐David 2015; Merkle, Potts & Fortin 2017). Furthermore, there are now a range of methods for detecting such history dependence in real‐world animal movement data (Börger, Dalziel & Fryxell 2008; Bar‐David *et al*. 2009; Riotte‐Lambert, Benhamou & Chamaillé‐Jammes 2013; Merkle, Fortin & Morales 2014; Riotte‐Lambert, Benhamou & Chamaillé‐Jammes 2017; Péron *et al*. 2016). Two of the mechanisms for generating history‐dependent movement include internal (cognitive) memory, and external (chemical) signals deposited into the environment. Indeed, considering their exceedingly small (<1 mm${}^{3}$) brains, rock ants exhibit impressive capacities for both internal (i.e. neuronal, McLeman, Pratt & Franks 2002; Stroeymeyt, Franks & Giurfa 2011; Bowens, Glatt & Pratt 2013), and external (i.e. pheromonal, Mallon & Franks 2000) memory storage formats. Therefore, the last part of this paper examines whether the trajectories of individual rock ants exhibit non‐Markovian properties, that is, whether movement decisions are history dependent.

## Materials and methods

### Study system

Twenty three *T. albipennis* ant colonies were collected on 3 May 2008 in Dorset, UK. They were housed in rectangular nests (internal dimensions: 50 × 35 × 2 mm), constructed by sandwiching a cardboard gasket between two glass microscope slides (Sendova‐Franks *et al*. 2010). Food and water were provided ad libitum. All colonies had a single queen that showed normal behaviour for a fertilized queen, for example a strong tendency to take up a position on the brood pile. Forty eight hours prior to the commencement of the experiments, all workers in each colony were individually tagged with a unique colour code of paint marks applied to the top of the head, the thorax, and the gaster. Immediately following the individual marking, the colonies were rehoused in a new nest with food and water provided ad libitum. Then, 24 h after the individual marking had ended 48 h of time‐lapse photography began. During this 48 h period, no food or water was provided to the colonies, although they were allowed access to an exploration arena (100 × 100 mm), which was accessible via a tube attached to the front of the nest. A digital camera captured an image of the nest once every 10 min.

From these images, we extracted 86 023 ant coordinates, comprising 335 ant trajectories. From each colony, we extracted the trajectory of the queen plus 14–16 randomly selected workers. Each trajectory consisted of a regular sequence of time‐stamped {*x*, *y*} coordinates, with a 10‐min interval between successive fixes. As ants sometimes left the nest, or were not visible within the nest, some coordinates were missing. These gaps accounted for a mean of 10·6 ± 0·6% of the trajectories, and the average trajectory consisted of 257 ± 2·5 time‐stamped coordinates.

Although the brood are not mobile, they are occasionally moved by the workers. Therefore, to map the slowly changing spatial distribution of the brood, several censuses were made over the course of each experiment; every 50 photographs (every 8 h) the developmental stage and {*x*, *y*} position of each brood item was recorded (Figs S1 and S2 in Appendix S1, Supporting Information). Each brood item was classified into four categories, according to developmental stage: (i) eggs, (ii) small larvae, (iii) medium/large larvae, or (iv) pre‐pupae and pupae (Franks & Sendova‐Franks 1992). For further details on the brood censuses, see the Appendix S1.

### Generating unbiased synthetic trajectories: a random walk null model

**Figure 1:**
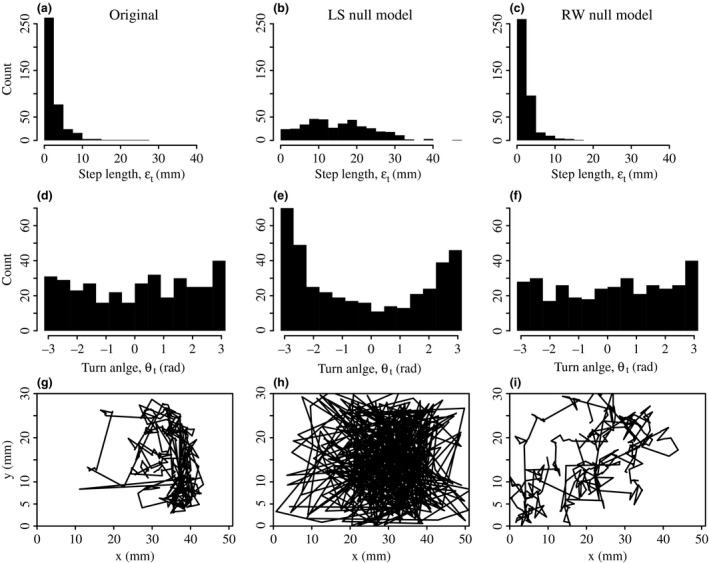
Producing unbiased synthetic ant trajectories. (a–c) The step‐length distributions is as follows: (a) ant 16 from colony 6, (b) a single realization of the Location‐shuffling null model, and (c) a single realization of the Random Walk null model. (d–f) The turn angle distributions. Notice that the LS null model does not preserve the shapes of either original distribution. (g–i) The trajectories corresponding to the original path, and the two null models. Notice the LS trajectory contains an over‐abundance of long‐range jumps and heading reversals, whereas the RW trajectory preserves the basic movement characteristics of the original.

In order to diagnose the presence of a non‐random pattern, one typically compares the observed pattern against an expectation or absolute standard produced by a null model. When testing for a non‐random movement pattern, such as site fidelity, the absolute standard is typically obtained by repeatedly randomizing the original trajectory, to obtain an ensemble of synthetic trajectories that are free from spatial biases and which can then be used as an absolute standard against which the original is compared. At least two previous studies on site fidelity in ants (Sendova‐Franks & Franks 1995) and bumblebees (Jandt & Dornhaus 2009) have used what we will call the Location Shuffling (LS) null model, in which a synthetic trajectory is constructed by randomly sampling coordinates from the trajectories of nestmates. However, because the LS procedure samples coordinates from the trajectories of multiple individuals, the resulting synthetic trajectories do not preserve two fundamental statistical properties of the original trajectory. The first of these fundamental properties is the step‐length distribution, where a step is defined as the distance between each coordinate, $\left(x_{t},y_{t}\right)$ and the next, $\left(x_{t+1},y_{t+1}\right)$, that is, $\varepsilon_{t}$ (Fig. 1a). The second is the turn‐angle distribution, where a turn is defined as the signed angular deviation $\theta_{t}$ (range −π to π) between successive line‐segments (Fig. 1d). As these distributions are not preserved (Fig. 1b,e), the synthetic LS trajectories exhibit several undesirable traits, for instance, they contain an over‐abundance of long‐range jumps and heading reversals (Fig. 1h).

Given these shortcomings, we require an alternative null model that produces unbiased synthetic trajectories that lack any spatial biases, but which also retain the fundamental statistical properties of the original. We therefore adopt a Random Walk (RW) null model that uses constrained randomization to produce synthetic trajectories that exhibit no spatial biases but also preserve these distributions (Munger 1984; Danielson & Swihart 1987; Spencer, Cameron & Swihart 1990; Schwarzkopf & Alford 2002; Richardson & Gorochowski 2015). The RW null model produces a synthetic trajectory by iterative random sampling (with replacement) from the step‐length and turn‐angle distributions of the original trajectory. This iterative sampling is stopped when the synthetic trajectory contains the same number of steps as the original. Because we use sampling with replacement, the synthetic step‐length and turn‐angle distributions for a single synthetic trajectory are not necessarily identical to the originals (Fig. 1c,f). Nevertheless, because many synthetic paths are produced for each original path, the aggregate synthetic distributions will converge to the originals.

In order to ensure that the only difference between the original and null model trajectories is the absence of spatial bias in the latter, three further constraints are imposed upon the above procedure. First, the synthetic trajectory is initiated at the same starting coordinates as the original. Second, to ensure that the synthetic path respects the internal borders of the nest, the iterative resampling is constrained: if, after any iteration, a random sample, $\left(\varepsilon_{t},\theta_{t}\right)$, takes the synthetic ‘ant’ beyond the internal boundary of the nest, then the sample is discarded and another drawn. Third, because the trajectories contained some gaps, the exact temporal gap structure of each ant trajectory was imposed upon the corresponding synthetic versions. The end result of the RW procedure is a synthetic trajectory that exhibits realistic movement patterns, but which lacks any localized spatial biases present within the original (Fig. 1i).

### Measuring site fidelity: a site‐centric approach

Many studies of site fidelity, particularly those focusing upon social insects, have adopted a reductive approach in which each trajectory is reduced to a single summary statistic, such as the area that it covers: an individual is classified as exhibiting site fidelity if its trajectory covers a significantly smaller area than a set of synthetic trajectories that exhibit no spatial bias, whereas it is classified as exhibiting ‘roaming’ if its trajectory covers a significantly larger area than the synthetic trajectories (Munger 1984; Danielson & Swihart 1987; Sendova‐Franks & Franks 1995; Jandt & Dornhaus 2009). Because this approach is based upon a comparison between the total area covered by the original and synthetic trajectories, it can fail to identify an individual that is attracted to a set of important sites but that nevertheless still covers a similar area to the corresponding synthetic trajectories. In Appendix S1, we present a simulation in which the area covered by a spatially biased random walk is compared with the area coverage expected in the absence of spatial biases (Fig. S3 in Appendix S1). This comparison showed no significant difference in the area covered by the spatially biased and unbiased walks, and thus demonstrates the validity of the claim that the reductive approach does not always correctly identify individuals that exhibit spatial bias.

The first stage of the site‐centric approach is to divide the study area into a regular grid. Here, we investigated two grid cell sizes, both on the order of the length of a single ant; 3 × 3 mm and 4 × 4 mm. As the results were very similar for both cell dimensions, in what follows we present only the results for the former.

**Figure 2:**
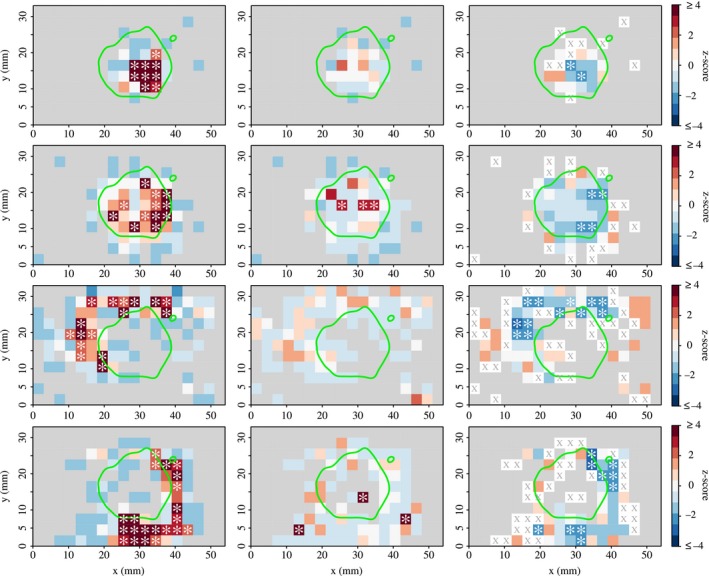
Testing for spatial fidelity at the level of individual sites. Each row of three panels represents a single ant from colony 6 (row 1; queen, rows 2–4; workers). The nest entrance is located midway along the *x*‐axis. The nest is divided into a regular 3 × 3 mm grid; cell colours indicate the magnitude and sign of the deviation of the observed number of visits ($n_{i}^{s}$, left column), the observed mean first return time ($\bar{{\text{RT}}_{i}^{s}}$, right column), or the observed mean dwell time ($\bar{D_{i}^{s}}$, middle column), from the corresponding null model expectation for that cell, expressed as standardized *z*‐scores. Grey cells indicate sites that the ant did not visit. Asterisks indicate sites whose observed value lay outside the distribution of values from the 10 000 synthetic trajectories. The green line indicates the edge of the brood pile. Sites that the ant visited only once (corresponding to censored return times) are indicated by an ‘X’.

In order to make comparisons between the reductive and site‐centric approaches, we first define a simple measure of the area that a trajectory covers; this is the number of unique sites that each ant *i* visits, $N_{i}$. Following the site‐centric approach of Boyer, Crofoot & Walsh (2011), for each individual we next define three measures for each site; the number of visits, the mean dwell time, and the mean first return time. A site visit is defined as an uninterrupted presence of a given individual at a given site. So for individual *i* and site *s*, the sequence of uninterrupted visits is, $V^{s}=\left\{v_{k=1},v_{k=2},...,v_{k=n_{i}^{s}}\right\}$, where the total number of visits is $n_{i}^{s}$ (Fig. 2, left column).

Each site visit $v_{k}$ also has an associated start and stop time, $v_{k}=\left\{v_{k}^{\mathrm{start}},\;v_{k}^{\mathrm{stop}}\right\}$. The dwell time for the *k*th visit is then $D_{v_{k}}^{s}=v_{k}^{\mathrm{stop}}-v_{k}^{\mathrm{start}}$, and the mean dwell time across the $n_{i}^{s}$ visits of individual *i* to site *s*, is, $\bar{D_{i}^{s}}$ (Fig. 2, middle column). A visit to a site that is later revisited also has an associated first return time, defined by the interval between the end of one visit and the beginning of the next. So if after the *k*th visit $v_{k}$, of individual *i* to site *s*, individual *i* returns to the same site, the first return time is, ${\text{RT}}_{v_{k}\to v_{k+1}}^{s}=v_{k+1}^{\mathrm{start}}\left(t\right)-v_{k}^{\mathrm{stop}}\left(t\right)$. However, as the first return times are defined by the intervals between successive visits, and as the observation period is finite, for each site *s* visited by ant *i*, the return time following the last visit, $n_{i}^{s}$, is unknown, or more properly, it is ‘censored’. Censoring complicates the estimation of an average first return time, for example, ignoring the censored returns and instead taking the mean across the uncensored return times induces a downward bias in the mean. Therefore, to obtain an unbiased estimate of the typical first return time for a given site *s*, we calculate the restricted mean (Irwin 1949), which we write $\bar{{\text{RT}}_{i}^{s}}$ (Fig. 2, right column).

To measure the extent to which a given site is ‘important’ to a given individual, it is necessary to compare the observed visit patterns with those expected under the null hypothesis that visits are random. To do so, for each site *s*, and each individual *i*, we compare the observed number of visits, $n_{i}^{s}$, the mean dwell time, $\bar{D_{i}^{s}}$, and mean first return time, $\bar{{\text{RT}}_{i}^{s}}$, with the corresponding distribution of expected values obtained from the RW synthetic trajectories. The three expected distributions for individual *i* at site *s* are obtained by first subjecting *i*'s trajectory to 10 000 null model randomizations, resulting in an ensemble of 10 000 synthetic trajectories, each based upon *i*'s original trajectory. While not all of the synthetic trajectories will visit site *s*, the distribution of the number of times that each synthetic trajectory visits *s* across the trajectory ensemble, gives the expected distribution for $n_{i}^{s}$. The expected distributions for the mean dwell time $\bar{D_{i}^{s}}$, and the mean first return time $\bar{{\text{RT}}_{i}^{s}}$, are obtained in the same way. To characterize the extent to which the observed individual site visitation patterns deviate from these expectations, the observed number of visits, the dwell and return times for each individual at each site were expressed as standardised *z*‐scores, $z=\frac{x-\mu }{\sigma }$, where *x* represents the observed value, μ represents the *mean* of the corresponding distribution of expected values, and σ represents the standard deviation thereof. We write these individual‐ and site‐specific *z*‐scores, as $n_{i}^{s}\left(z\right)$, $\bar{D_{i}^{s}}\left(z\right)$, and $\bar{{\text{RT}}_{i}^{s}}\left(z\right)$. Finally, to identify the important sites for individual *i*, for each of the sites that it visits we test the null hypothesis that the observed number of visits $n_{i}^{s}$, mean dwell time, $\bar{D_{i}^{s}}$, or mean first return time, $\bar{{\text{RT}}_{i}^{s}}$, are statistically indistinguishable from the corresponding distributions of expected values produced by the RW null model. As there are no particular *a priori* reasons to predict whether ants should be biased towards or away from particular sites, for each site *s* visited by ant *i* we perform a two‐tailed permutation test using a significance threshold of α < 0·05. So in the case of the number of visits to a given site, $n_{i}^{s}$, the null hypothesis is rejected if the observed $n_{i}^{s}$ is lower than the leftmost 0·025 quantile of the expected distribution, on the grounds that the ant made significantly fewer visits to *s* than expected. Similarly, the null hypothesis is also rejected if the observed $n_{i}^{s}$ is greater than the rightmost 0·975 quantile of the expected distribution, because the ant visited *s* significantly more than expected. However, as each ant *i* visits $N_{i}$ different sites, there are $N_{i}$ significance tests for each ant *i*, hence it is likely some sites may achieve a statistically significant *P*‐value just by chance. Therefore, to control for the effect of multiple comparisons, we apply the Benjamini–Hochberg false discovery rate procedure (Benjamini & Hochberg 1995) to the two‐tailed *P*‐values. After this correction, any remaining sites with *P* < 0·05 are classified as important.

Lastly, because one of the later analyses requires that we compare between different groups of individuals, we here define three individual‐level summary statistics corresponding to the site‐centric measures, namely, the mean number of visits per visited sites $\bar{n_{i}}$, the mean dwell time per visited site, $\bar{D_{i}}$, and, the mean return time per visited site, $\bar{{\text{RT}}_{i}}$.

### Distinguishing communities with distinctive patterns of site fidelity

**Figure 3:**
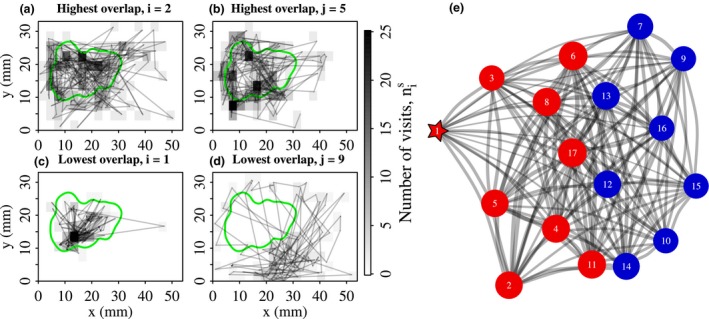
Classifying ants into groups with distinctive space use patterns. This figure illustrates the procedure for colony 1. (a, b) Trajectories of the two ants with the most similar space use patterns. Site greyscale indicates the number of times the ant visited each site, $n_{i}^{s}$. The spatial overlap between $n_{i=2}^{s}$, and $n_{i=4}^{s}$, is VI${}_{i,j}\;=\;0\cdot 66$. The green line indicates the edge of the brood pile. (c, d) Trajectories of the two ants with the most dissimilar space use patterns, which have VI${}_{i,j}\;=\;0\cdot 03$. Note, ant *i* = 1 is the queen. (e) Network representation of the spatial relationships between ants. Edge widths are proportional to the magnitude of the pairwise spatial overlap, VI${}_{i,j}$. Vertex size is proportional to the weighted degree centrality. The queen is indicated by the star. Vertices are coloured according to their community membership. For this colony, two communities were detected. Red nodes – ants in the queen community, labelled ‘nurses’. Blue nodes – ants in the second community, labelled ‘other’.

Here we describe how the site‐centric approach can be used to classify individuals into groups with distinctive space use patterns. The process involves three steps. In the first step, for each unique pair of individuals, {*i*, *j*}, we use the number of visits that each ant makes to each site ($n_{i}^{s}$, $n_{j}^{s}$) to calculate a well‐established measure of spatial overlap, namely the volume of intersection (Kernohan & Gitzen 2001; Fieberg & Kochanny 2005). When *i* and *j* visit each site exactly the same number of times, then the spatial overlap is maximal, and the volume of intersection VI${}_{i,j}$ = 1 (Fig. 3a,b). Conversely, when *i* visits none of the sites visited by *j* then there is no overlap, and hence VI${}_{i,j}$ = 0 (Fig. 3c,d).

In the second step, these pairwise spatial overlaps are used to construct a network in which each individual *i* is represented by a vertex, and each pair correlation, VI${}_{i,j}$, is assigned as the weight of an undirected edge connecting nodes *i* and *j* (Fig. 3e). To identify groups of individuals with similar space use patterns, we apply the Spinglass community‐detection method (Reichardt & Bornholdt 2006). All network analyses were conducted, using the igraph package (version 1.0.1; Csardi & Nepusz 2006) for R.

**Figure 4:**
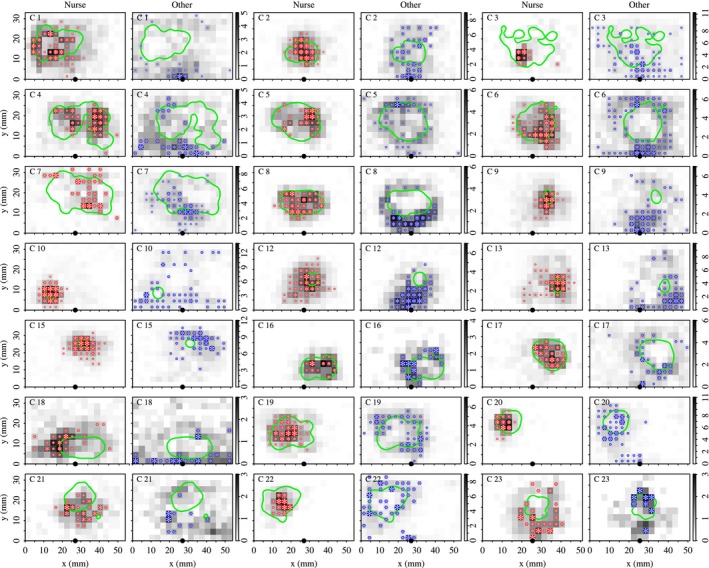
Within‐community space use maps for the 21 colonies that had two communities. Site greyscale indicates the number of times ant *i* visited the site, $n_{i}^{s}$, averaged across all community members. Asterisks indicate important sites for community members; those that received significantly more visits than the null model expectation. Asterisk size is proportional to the number of community members for which the site was classified important. The black point on the *x*‐axis indicates the nest entrance. The green line indicates the edge of the brood pile.

As the network is essentially a topological representation of spatial relationships between individuals, in the last step, the space use maps of the different communities were compared. To do so, within‐community averages were calculated for the number of visits to each site, $n_{i}^{s}$, thus producing a map of site visitation frequencies that is representative of the community (Fig. 4).

### Detecting the statistical signature of self‐reinforcing spatial bias

We now outline a further elaboration of the site‐centric approach, in which we test whether ant movement decisions display the statistical characteristics associated with a particular movement model that has been suggested as a likely candidate for producing site fidelity, that is, the self‐attracting walk (Tan *et al*. 2001; Börger, Dalziel & Fryxell 2008). Because an agent that performs a self‐attracting walk is attracted towards locations that it visited in the past, we here explore whether individuals return to the sites that they have previously visited more rapidly than expected by random chance.

We follow the general approach of Boyer, Crofoot & Walsh (2011) and test whether the time an individual *i* dwells at site, *s*, during its *v*th visit, $D_{v}^{s}$, predicts the time it takes until it *next* visits site *s*, which is the first return time, ${\text{RT}}_{v\to v+1}^{s}$. The null hypothesis that ants move completely randomly within the nest can be rejected if ${\text{RT}}_{v\to v+1}^{s}$ exhibits a statistically significant dependence upon $D_{v}^{s}$. A negative relationship – obtained when long dwell times are associated with short return times – is indicative of a self‐attracting walk in which previously visited sites become more attractive with each visit (Tan *et al*. 2001; Foster, Grassberger & Paczuski 2009). Conversely, a positive relationship indicates a self‐avoiding walk in which intensively‐visited sites are avoided (Madras & Slade 1993; Richardson *et al*. 2011). In testing the null hypothesis of no relation between $D_{v}^{s}$ and ${\text{RT}}_{v\to v+1}^{s}$, Boyer, Crofoot & Walsh (2011) pooled all site‐visits across all individuals, discarded all sites that received less than five visits, and then used linear least‐squares regression to test whether the dwell time predicted the return time. Here, however, we use an alternative method to address the same question; a Cox survival model (Cox 1972), with a mixed‐effects extension that allows variation arising from uncontrolled variables to be included as random effects (Therneau 2000). Survival modelling was conducted using the package coxme (version 2.2‐5) for R. We use this method because it allows the inclusion of censored time‐to‐event response variables, such as the unobserved return time that follows the last visit of individual *i* to site *s*, that is, ${\text{RT}}_{v_{n}\to v_{n+1}}^{s}$, and also because the mixed‐effects extensions renders it robust to uncontrolled variation between individuals and colonies.

As in Boyer, Crofoot & Walsh (2011), the response was the time to return to a site following the end of the *v*th visit, ${\text{RT}}_{v\to v+1}^{s}$, and the predictor was the dwell time during the *v*th visit, $D_{v}^{s}$. Furthermore, because the return time might have also been influenced by both the physiological or behavioural characteristics of the individual, or by other environmental features, we included three additional predictors in the model. These were, respectively, the reproductive caste of ant *i* (queen or worker), the community membership of *i*, as defined by the network partitioning, and the number of brood items (eggs, small larvae, large larvae, pupae) at site *s* during the *v*th visit of ant *i*.

The mixed‐effects extension allowed the following uncontrolled variables to be coded as categorical random effects, namely the identity of (i) each colony (21 levels, *A*, *B*, …, *Y*), (ii) each individual (335 levels, $A_{1},\;A_{2},\;\ldots ,\;Y_{13},\;Y_{14}$), and (iii) each site (198 levels). From these three random effects, we defined three candidate models. First, the maximal model that included all three random effects. The intermediate model retained colony and individual identity, but discarded the random effect with the greatest number of levels, namely, ‘site’. Finally, the minimal model retained the random effect with the fewest levels, namely colony identity. We used AIC to select among the three competing survival models; the model with the lowest AIC was the intermediate model, so we present and interpret the results from that model (Table 1). The results of all three models were qualitatively similar.

**Table 1:** Mixed‐effects survival model, testing how the time an ant *i* takes to return to a site *s* after the end of the *v*th visit, ${\text{RT}}_{v\to v+1}^{s}$, is influenced by (i) the duration of the *v*th visit, that is, the dwell time $D_{v}^{s}$, (ii) the reproductive caste of the ant, (iii) the community to which it belongs, and (iv) the spatial distribution of brood of different developmental stages

Predictor	HR	SE	*z*	*P*
Dwell time, $D_{v}^{s}$	1·07	0·00295	21·9	***
Community	1·3	0·0327	7·94	***
Reproductive caste	1·91	0·0607	10·6	***
N eggs at site	1·02	0·00271	8·06	***
N small larvae at site	1·03	0·00578	4·27	***
N large larvae at site	1·04	0·00825	4·51	***
N pupae at site	1·17	0·013	11·8	***

For non‐categorical predictors, the hazard ratio (HR) indicates the instantaneous risk of a return visit to *s*, relative to the baseline hazard. For the categorical predictors, caste and community, the HR indicates respectively, the instantaneous risk that a queen returns to site *s* relative to a worker, and the instantaneous risk that an ant in the ‘nurse’ community returns to *s*, relative to an ant in the ‘other’ community. Colony and ant identity were coded as random factors, with ant identity nested within colony identity. Two colonies were excluded from the analysis because their spatial interaction network had only 1 community, hence ants could not be labelled according to their community. The model was based upon 50 187 site visits, of which 29 391 were uncensored site‐returns. The ‘***’ indicates *P* $<10^{-4}$.

## Results

### Superiority of the site‐centric approach

The presence of spatial fidelity was strongly supported by both the traditional reductive approach and the combined site‐centric approach. Out of the 335 ant trajectories, the reductive approach found that no ants visited a larger area than expected (no roaming), whereas 152 ants visited a significantly smaller area than expected (site fidelity). All of the remaining 183 ants visited an area that was no larger or smaller than expected.

Among 152 ants that the reductive approach classified as exhibiting fidelity, the site‐centric approach found 133 that visited at least one site significantly more than expected (site fidelity), whereas 18 visited at least one site less than expected (site avoidance). Therefore, by and large, the two approaches agreed over the identity of the ants that exhibit site fidelity. However, among the 183 ants that the reductive approach classified as exhibiting no spatial bias, the site‐centric approach found 57 that visited at least one site significantly more than expected (site fidelity), whereas 24 visited at least one site less than expected (site avoidance). Because 81 of the 183 ants that the reductive approach classed as exhibiting no spatial bias did in fact exhibit bias, we conclude that the site‐centric approach is considerably more sensitive.

There is at least one additional reason to prefer the site‐centric approach over the reductive approach: because the reductive approach reduces the trajectory to a single statistic, such as the area covered, it can only assign an individual to one of three categories, namely, site fidelity (area smaller than expectation), neutral (area equal to expectation), or ‘roaming’ (area larger than expectation). The site‐centric approach provides a richer classification scheme, as in addition to the above three categories, it can also identify individuals that are attracted to some sites whilst also avoiding other sites. Furthermore, by expressing the site visits statistics as standardized z‐scores, it can provide information about the magnitude and direction of the bias to particular sites (Fig. 2).

We now summarize the extent of site fidelity, as measured by the site‐centric approach. All three site‐centric measures of fidelity, $n_{i}^{s}$, $\bar{D_{i}^{s}}$ and $\bar{{\text{RT}}_{i}^{s}}$, showed considerable variation in the extent to which ants exhibit fidelity to particular sites; the mean number of important sites per ant was 8·5 ± 0·30 for $n_{i}^{s}$, 3·3 ± 0·17 for $\bar{{\text{RT}}_{i}^{s}}$, and 1·5 ± 0·1 for $\bar{D_{i}^{s}}$. However, across the three metrics, there was an appreciable degree of consistency in the identity of the important sites (see asterisks in Fig. 2).

Visual inspection showed that across all 23 colonies, the important sites of the queen were almost invariably restricted to sites containing many brood items (Fig. 2, row 1). However, those of the workers either clustered around the queen and brood (Fig. 2, row 2), or formed more peripheral shapes, such as sickle‐shaped formations around the border of the brood pile, or tight clusters around the nest entrance (Fig. 2, rows 3–4).

### Ants can be classified into communities with distinctive site fidelity patterns

We now outline the results of the social network analysis of the spatial overlaps between ant pairs. One of the most obvious low‐level features of the spatial networks was the clear differences between the workers and the queens: even though queen movement was very biased towards the biological centre of the nest (i.e. the brood pile; Fig. 3c), this spatial centrality did not translate into topological centrality, as queens instead occupied peripheral positions on the spatial networks (Figs 3e, S4). To quantify this observation, we measured the weighted degree centrality of each vertex in each network: high values indicate ants whose important sites are shared with many other ants, whereas lower values indicate ants whose important sites are shared with few others. Compared to workers, queens had a significantly lower weighted degree (*Q* vs *W*;* t* = −3·6, *P* = 0·0003). Therefore, the site‐centric approach revealed that despite outward appearances, queens were spatially isolated from workers.

The overall structure of the interaction networks was remarkably consistent across colonies: 21 of the 23 spatial networks were partitioned into two communities, whilst two networks could not be partitioned (Fig. S4 in Appendix S1). A universal feature of behavioural organization in the social insects, is that young workers tend to feed and groom the brood and the queen, whereas older workers perform generalist within‐nest tasks, and the oldest workers guard the nest entrance and go outside to forage (Oster & Wilson 1978; Seeley 1982; Mersch, Crespi & Keller 2013). Therefore, in the two‐community colonies the identity of the community containing the queen provided a convenient means of applying a biological meaningful label to each community: the community containing the queen was labelled ‘nurses’ (N), and the remaining community was labelled ‘others’ (O).

In the two community colonies, the movement patterns of the ants varied according to the identity of their community. In the 21 two‐community colonies, the ants in the ‘nurse’ community visited significantly fewer sites (linear mixed‐effects regression, response; $N_{i}$, predictor; caste, random effect; colony identity, $F_{1,288}\;=\;115\cdot 0$, *P* < 10${}^{-4}$, henceforth ‘${}^{***}$’), made significantly more visits to each of the sites that they did visit (response $\bar{n_{i}}$; $F_{1,295}\;=\;59\cdot 2$, *P* = ${}^{***}$), had a significantly longer site dwell time (response $\bar{D_{i}}$; $F_{2,289}\;=\;31\cdot 7$, *P* = ${}^{***}$), and returned to previously‐visited sites significantly more rapidly (response $\bar{{\text{RT}}_{i}}$; $F_{1,297}\;=\;73\cdot 3$, *P* = ${}^{***}$) than ants in the ‘other’ community. So, nurse workers exhibited a much greater degree of spatial fidelity than the other workers.

Converting the network partitions into within‐community space use maps, revealed that the topologically distinct communities corresponded to groups with quite distinct space use patterns. The nurse community tended to visit sites within the brood pile and only rarely visited sites outside the brood pile (Fig. 4). In the 21 colonies with two communities, the second community (which we labelled ‘other’) tended to avoid sites at the centre of the brood pile, and instead occupied positions at its edge, and also around the nest entrance (Fig. 4).

### Ants exhibit self‐reinforcing spatial bias

The survival model confirmed that ant movement displays biased return statistics that are not consistent with a Markovian movement model: each additional minute that an ant spent at a given site before departing, modified the instantaneous risk of a return by 1·07‐fold over the baseline hazard rate, which is an increase of 7% per minute (Table 1). Therefore, short visits were associated with long waits until the next visit, whereas long visits were associated with short waits until the next visit. This association is consistent with a self‐attracting walk, a movement model in which visited sites become more attractive with each visit. Because in this model, previous site‐visits influence future behaviour, self‐attracting walks are history dependent, or more properly, they are non‐Markovian.

Several other factors also influenced the time taken to return to a previously visited site: the effect of an ant belonging to the ‘nurse’ community was to modify its instantaneous return risk by 1·3‐fold relative to those ants in the ‘other’ community. This confirms the result from the between‐community comparisons in the previous section. However, the reproductive caste of the ant was by far the strongest determinant of the return time: the effect of an ant being a queen was to increase its instantaneous return risk by 1·9‐fold, that is, a 90% increase relative to a worker. Thus, queens more rapidly returned to previously‐visited sites than workers. The presence of brood items at a site did significantly influence the instantaneous risk that the individual would return there. However, the direction of the effect was dependent upon the developmental stage of the brood item. Each additional egg at a site was associated with a 1·02‐fold increase in the instantaneous return risk over the baseline hazard rate. Similarly, each additional small larva, large larva and pupa were associated with a 1·03, 1·04, and 1·17‐fold respective increase in the return risk. Therefore, all brood stages were attractive to the ants, and more the brood were present at a given site during the visit of an ant, the more rapidly the ant returned there.

## Discussion

In this paper, we have presented an analytical framework that leverages the spatial and temporal information contained within an animal trajectory to identify important sites within the environment, identify groups of animals with distinctive space‐use patterns, and shed light on the mechanisms that underpin animal movement. We have also shown that this combined framework is considerably more sensitive than previous approaches which reduce a complex spatio‐temporal object – an animal trajectory – to a single summary statistic. This sensitivity is derived from the formal statistical hypothesis testing provided by comparisons between the original trajectories and the synthetic trajectories produced by the RW null model. It should be emphasized that this null model could profitably be combined with other site‐specific methods for quantifying local space‐use intensities, such as those of Benhamou & Riotte‐Lambert (2012) and Lyons, Turner & Getz (2013), to identify locations that are more intensively exploited or more frequently revisited than expected by chance alone.

In addition to these methodological results, our application of the above combined framework to within‐nest ant trajectories also provided several novel biological conclusions. The first concerns the theory of ‘organisational immunity’ (Schmid‐Hempel & Schmid‐Hempel 1993; Stroeymeyt, Casillas‐Pérez & Cremer 2014), which predicts that animal societies in which there is a reproductive division of labour, should possess structural features – such as bottlenecks, or compartmentalization – that inhibit transmission of pathogens to the reproductive individuals. The spatial network analysis provides two lines of support for the presence of organizational immunity in *T. albipennis*. The first was that 21 of 23 colonies were segregated into two groups, with the group that contained the queen always being the group that was found closest to the biological centre of the colony, that is, the brood pile. The second was that even though queens were typically found at the centre of the nest, the sites to which they exhibited bias overlapped little with those of most workers, which led to them occupying peripheral positions on the spatial network. Thus, queens were spatially and socially isolated from the workers. In social insect colonies, it is typically the outside‐nest workers that are most likely to expose the colony to risk, for example, by bringing back a pathogen after a foraging trip outside the nest (Schmid‐Hempel & Schmid‐Hempel 1993). As here, the ‘other’ group overlapped little with the brood pile, and was instead concentrated around the nest entrance, it is likely that this group contained many such outside‐nest workers. Therefore, the compartmentalization of the colony into layered groups and the isolation of the queen within the innermost group could be interpreted as organizational features that reduce the exposure of the colony to pathogens.

The second conclusion concerns the mechanisms responsible for generating site fidelity. Although the presence of site fidelity is well documented across a range of social insect species, to the best of our knowledge nothing is known about how individuals first establish and then maintain bias towards a set of important sites. The finding that the longer an ant dwells at a site the more quickly it will return after leaving it, indicates that ant movement is not compatible with a Markov movement model, or in other words, ‘history’ influences current behaviour. The observed statistical signatures appear consistent with a particular class of non‐Markov movement model, the so‐called self‐attracting walk, in which sites become progressively more attractive with each visit. Indeed, the self‐attracting walk has been proposed as a candidate mechanism that would allow an animal to establish and maintain fidelity towards a set of important sites (Tan *et al*. 2001; Foster, Grassberger & Paczuski 2009). However, it is important to note two caveats. First, this association is a correlation, so it cannot be claimed that the long site dwell times cause short returns. Second, whilst the presence of a statistical signature of a self‐attracting walk indicates that this may underly the generation of site fidelity in *T. albipennis* ants, this result does not say anything about the nature of the ‘memory’ that allows the reinforcement to be brought about. Nevertheless, there are at least two (potentially complimentary) candidate mechanisms, namely, chemical pheromones deposited onto the substrate, and internal place memory. As rock ants have evolved sophisticated strategies for chemical marking (Mallon & Franks 2000), and navigation strategies (McLeman, Pratt & Franks 2002; Bowens, Glatt & Pratt 2013), future research should concentrate on elucidating their contributions to the generation of site fidelity.

In this paper, we have outlined a combined framework for identifying the sites to which individuals are attracted, and for identifying groups of individuals that share a common set of sites to which they are attracted. We hope that the clear advantages of the site‐centric framework over traditional reductive approaches, will encourage others to delve further into the mechanisms that govern animal movement.

## Authors’ contributions

T.O.R., A.B.S.‐F. and N.R.F. conceived the experiments. T.O.R., L.G., A.B.S.‐F. and N.R.F. conceived the analyses. T.O.R. performed the experiments and the analysis. T.O.R. wrote the paper with input from all authors. N.R.F. and A.B.S.‐F. provided experimental facilities. All authors commented and provided feedback on the manuscript.

## Data accessibility

Ant trajectories and brood location data are freely available from the Dryad Digital Repository at https://doi.org/10.5061/dryad.fj043 (Richardson *et al*. 2017).

## Supporting information


**Appendix S1.** This document (i) contains information about the colony demographics, (ii) describes simulations that compare the reductive and site‐centric approaches to measuring site fidelity and (iii) shows the spatial networks for all 23 colonies.
