# Supplementary material for: Measuring site fidelity and spatial segregation within animal societies
Source: Methods Ecol Evol. 2017 Mar 20;8(8):965–75. doi: 10.1111/2041-210X.12751 (PMC5586202; doi:10.1111/2041-210X.12751)
Supplement: Supplementary file 1 — Appendix S1. This document (i) contains information about the colony demographics, (ii) describes simulations that compare the reductive and site‐centric approaches to measuring site fidelity and (iii) shows the spatial networks for all 23 colonies. [file MEE3-8-965-s001.pdf]

Measuring site fidelity and spatial segregation within animal societies: Supporting information

Thomas O. Richardson, Luca Giuggioli, Nigel R. Franks, Ana B. Sendova-Franks

Tracking the brood

Although the brood may appear stationary, the workers do move them, so it is necessary to occasionally update the brood item locations occasionally to compensate for their slow movement. To do so, in the first photograph, and every 50 photographs (i.e. every 500 minutes) thereafter, a census of the brood was taken. This involved manually recording of the location and developmental stage of each item (Fig. S1). An example of the spatial distribution of the brood over time is shown in Figure S2, and the censuses demographics are summarized in (Table. S1)

In order to obtain the number of brood items in each site s at each time-point t, the ant trajectories were split according to the nearest brood census. Thus, trajectory coordinates occurring in the range $1<t<25$ were assigned to the first census (at t=1), and those occurring in the range $26<t<75$ were assigned to the second census (at t=51), and so on. Hence the brood locations were never more than 25 photographs out of date.

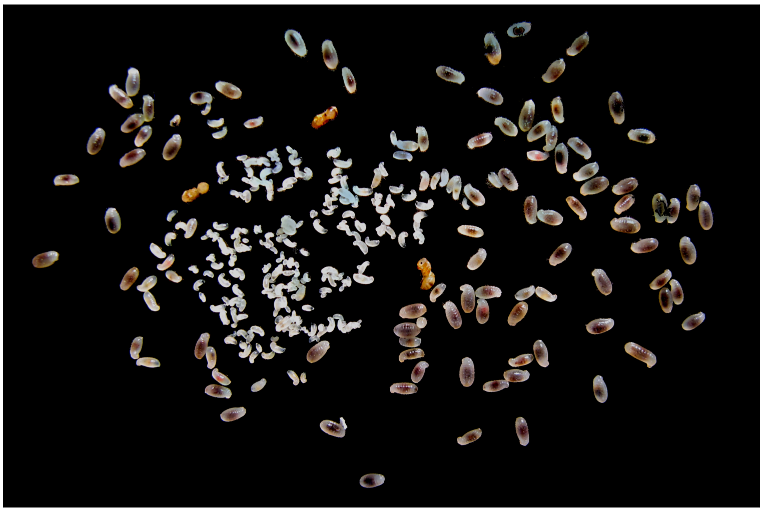

Figure S1: Example photograph of the brood pile. The small white items in central positions are eggs and small larvae. The grey peripheral items are large larvae. The orange items are pupae.

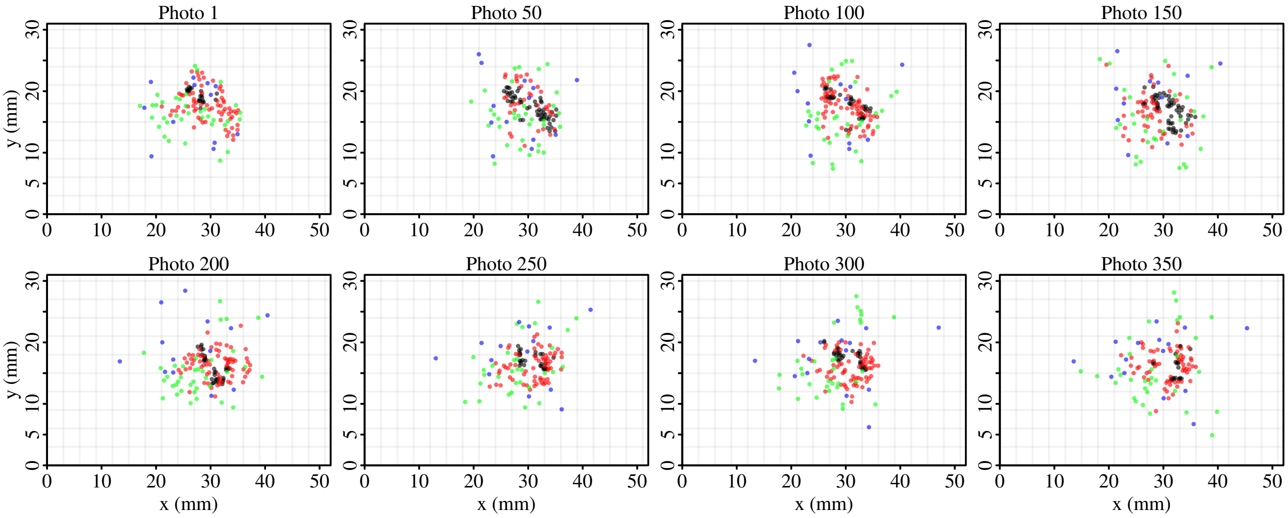

Figure S2: Censusing the brood. Each panels shows a single brood census from colony 6. Black, red, green and blue points respectively represent eggs, small larvae, large larvae & pupae.

Table S1: Colony-level summary of brood and adult demographics. Brood numbers represent means calculated across the brood censuses, rounded to the nearest whole number. Brood censuses were carried out every 50 photos (i.e. approximately every 8h). All colonies were queenright.

**Colony**	**N eggs**	**N small larvae**	**N large larvae**	**N pupae**	**N adults**
1	29	38	40	27	71
2	8	5	8	22	59
3	30	66	33	5	78
4	53	34	56	0	118
5	64	67	66	0	96
6	36	59	32	13	95
7	110	69	72	39	88
8	26	27	28	21	93
9	6	0	0	0	134
10	7	0	0	0	90
11	0	0	0	0	61
12	12	0	0	0	67
13	6	0	0	0	79
14	3	0	0	0	47
15	4	0	0	0	76
16	31	24	29	1	67
17	26	39	42	1	60
18	42	15	29	0	90
19	73	17	23	26	90
20	16	8	7	15	115
21	12	14	39	2	96
22	58	25	37	3	80
23	13	20	13	1	64

Reductive versus site-centric approaches to site fidelity

In the 'traditional' reductive approach to detecting site fidelity, each trajectory is reduced to a single summary statistic, such as the area that it fills. If the observed trajectory fills a significantly smaller area than do synthetic trajectories produced by a null model, the individual is then classified as exhibiting site fidelity, whereas if the observed area is larger than the sythetic trajectories, then the individual is classified as exhibiting 'roaming' behaviour (Munger, 1984; Danielson and Swihart, 1987; Sendova-Franks and Franks, 1995; Jandt and Dornhaus, 2009).

The reductive approach rests upon a comparison between the area covered by the original and synthetic trajectories. In this section we show that because the reductive approach focuses on the scale of the range used by an individual, it is not a particularly good choice for identifying an individual that is attracted to one or several sites, but but whose trajectory nevertheless still covers a similar area to that covered by a set of synthetic trajectories that by design exhibit no spatial biases. Other methods are needed to identify site fidelity at smaller spatial scales.

We here present a simple demonstration of this. First, we define a regular grid of sites towards which an agent will be attracted. Using this array, we then simulate a biased random walk in which the bias towards a given attractive site is dependent upon the distance from the site (Fig. S3a). This model is also known as the Ornstein-Uhlenbeck process (Preisler et al., 2013). We then define a square grid (with arbitrary dimensions), and count the number of sites (i.e. grid squares) that the agent visits. Here, N_i=1398. Next, an unbiased version of the original trajectory is generated using the Random Walk null model (Fig. S3b). The RW null model is repeated 1000 times, and the number of sites that each synthetic RW trajectory covers is recorded. Finally, we test the null hypothesis that the number of sites that the agent was observed to visit belongs to the distribution of 1000 N_i values from the synthetic trajectories. In the present case, this null hypothesis cannot be rejected (p=n.s.). The failure of the reductive approach to reject the null hypothesis leads to the erroneous conclusion that the original trajectory exhibits no spatial bias.

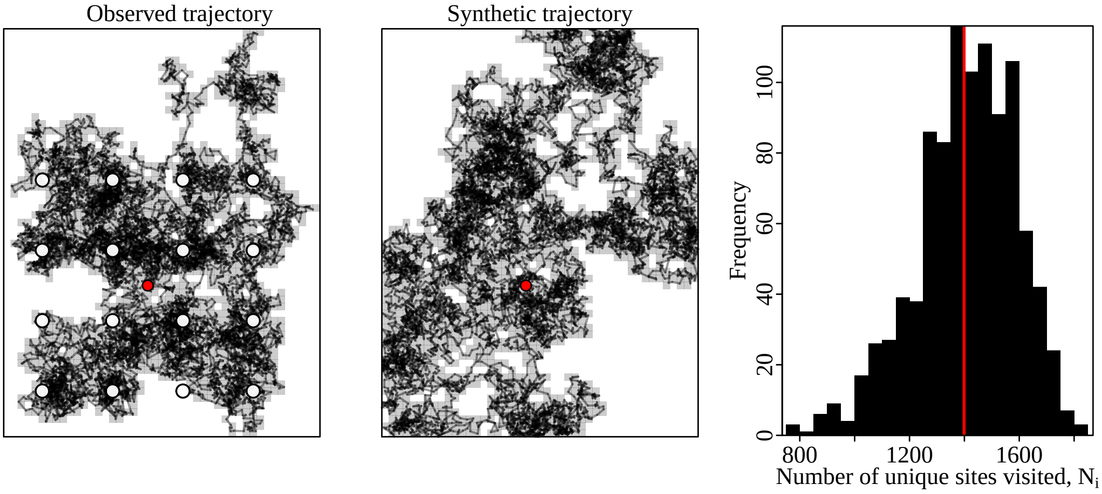

Figure S3: The reductive approach is not an optimal choice for identifying attractive sites. (**a**) Trajectory of an agent i, that is attracted to multiple locations (white points). Grey background shading indicates the area (number of sites) covered by the trajectory, N_i. The red dot indicates the starting location. (**b**) Synthetic version of the original trajectory, produced by the Random Walk null model. Notice that the trajectory is no longer biased towards any locations. (**c**) Comparison between the number of sites visited by the original trajectory (red vertical line), and an ensemble of 1000 synthetic trajectories produced by the null model (black bars). Notice that the number of sites visited by the original trajectory is indistinguishable from the number visited by the synthetc trajectories, hence the reductive approach fails to detect the expression of site fidelity.

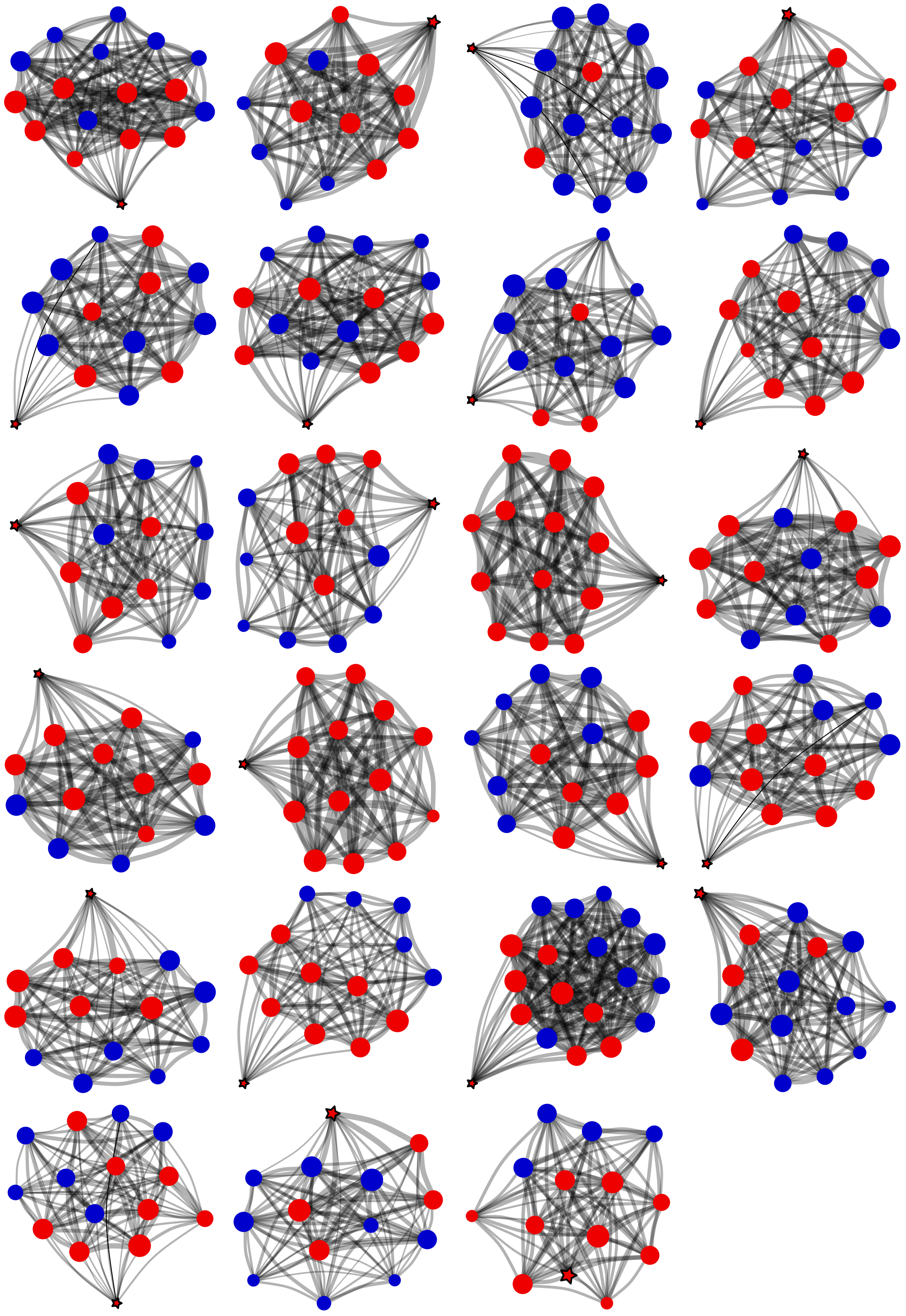

Figure S4: Network representation of the spatial (dis)similarities for all 23 colonies. Edge widths are proportional to the spatial overlap, $\mathrm{VI}_{i,j}$. The queen is indicated by the star. Vertices are coloured according to their community membership. Vertex size is proportional to the weighted degree. Red nodes - the queen community, labelled 'nurses'. Blue nodes - the 'other' community.

References

Danielson, B. J. and R. K. Swihart (1987). Home range dynamics and activity patterns of Microtus ochrogaster and Synaptomys cooperi in syntopy. *Journal of Mammalogy 68*(1), 160–165.

Jandt, J. M. and A. Dornhaus (2009). Spatial organization and division of labour in the bumblebee Bombus impatiens. *Animal Behaviour 77*(3), 641–651.

Munger, J. C. (1984). Home ranges of horned lizards (Phrynosoma): circumscribed and exclusive? *Oecologia 62*(3), 351–360.

Preisler, H. K., A. A. Ager, and M. J. Wisdom (2013). Analyzing animal movement patterns using potential functions. *Ecosphere 4*(3), 1–13.

Sendova-Franks, A. and N. Franks (1995). Spatial relationships within nests of the ant Leptothorax unifasciatus (Latr.) and their implications for the division of labour. *Animal Behaviour 50*(1), 121–136.
